# Identification of the Best Cut-Off Value of PIVKA-II for the Surveillance of Patients at Risk of Hepatocellular Carcinoma Development

**DOI:** 10.3390/biology12010094

**Published:** 2023-01-07

**Authors:** Gian Paolo Caviglia, Maria Lorena Abate, Giulia Troshina, Patrizia Carucci, Emanuela Rolle, Alessandra Risso, Michela Emma Burlone, Alice Albè, Martina Crevola, Emma Clara Musso, Chiara Rosso, Angelo Armandi, Antonella Olivero, Rosalba Minisini, Giorgio Maria Saracco, Elisabetta Bugianesi, Mario Pirisi, Alessia Ciancio, Silvia Gaia

**Affiliations:** 1 Department of Medical Sciences, University of Turin, 10126 Torino, Italy; 2 Gastroenterology U, Città della Salute e della Scienza—Molinette Hospital, 10126 Torino, Italy; 3 Department of Translational Medicine, Università del Piemonte Orientale, 28100 Novara, Italy; 4 I. Department of Medicine, University Medical Center of the Johannes Gutenberg-University, 55131 Mainz, Germany; 5 Department of Internal Medicine, “AOU Maggiore della Carità”, 28100 Novara, Italy

**Keywords:** biomarkers, HCC, protein induced by vitamin K absence or antagonist II

## Abstract

**Simple Summary:**

The use of tumor biomarkers, although debated, for the surveillance of patients at risk of HCC, is common practice in many clinical centers. Here, we investigated the best cut-off value for one of such biomarkers, protein induced by vitamin K absence, or antagonist-II in a large cohort of patients with liver cirrhosis under surveillance. We observed that a cut-off value of 50 mAU/mL was appropriate for the discrimination between patients with hepatocellular carcinoma and those without tumor, and among the latter, allowed the identification of patients with an increased risk of developing hepatocellular carcinoma during the follow-up.

**Abstract:**

Patients with cirrhosis are at risk of hepatocellular carcinoma (HCC) development and, according to current guidelines, should undergo surveillance by ultrasound at six month intervals. Due to the known limitations of surveillance strategies based on ultrasonography, the use of tumor biomarkers, although debated, is common practice in many centers. The aim of the study was to identify the best cut-off value for one of such biomarkers, protein induced by vitamin K absence, or antagonist-II (PIVKA-II). We retrospectively enrolled 1187 patients with liver cirrhosis: 205 with a diagnosis of HCC (median age 67 years, 81.0% males) and 982 without tumor (median age 64 years, 56.2% males). During a median follow-up (FU) of 34.6 (11.4–43.7) months, 118 out of 982 (12.0%) patients developed HCC. Serum PIVKA-II was assessed by chemiluminescence immunoassay on the Lumipulse^®^ G600 II platform (Fujirebio, Tokyo, Japan). In the overall cohort (*n* = 1187), PIVKA-II showed an area under the curve (AUC) of 0.802 for HCC detection. The best cut-off value that maximized sensitivity was 50 mAU/mL (sensitivity = 80%, specificity = 64%). In the 982 patients without HCC at baseline, PIVKA-II > 50 mAU/mL was associated with an increased risk of HCC development during the FU (HR = 1.74, 95% CI 1.21–2.51; *p* = 0.003)). In conclusion, the evaluation of serum PIVKA-II showed a good performance for HCC detection; a cut-off value > 50 mAU/mL could be suitable for the surveillance of patients who are at risk of developing HCC.

## 1. Introduction

Patients with liver cirrhosis should undergo surveillance due to the risk of hepatocellular carcinoma (HCC) development [1]. To date, HCC accounts for approximately 90% of all primary liver cancers, with a significant medical impact worldwide [2]. Though chronic viral infections represent the main cause of chronic liver diseases and cirrhosis [3,4], in the last decades the raising burden of non-alcoholic fatty liver disease has emerged as a substantial determinant of liver cirrhosis and its complications, including HCC [5,6]. To date, abdominal ultrasonography (US) represents the gold standard surveillance tool for the detection of HCC in high-risk populations, while the use of serum biomarkers in this setting is still up for dispute [7]. However, given US suboptimal accuracy for early HCC detection [8], and considering the low rates of patients’ retention to surveillance [4,9], the role of serum biomarkers in novel surveillance strategies is worth attention.

Alpha-fetoprotein (AFP) has been extensively investigated as a biomarker for HCC detection; however, due to its suboptimal performance in terms of sensitivity (Se) (AFP-negative HCCs) [10], and its poor specificity (Sp) [11], there is no consensus on the use of AFP for the surveillance of patients who are at risk of developing HCC. Protein induced by vitamin K absence or antagonist-II (PIVKA-II), an aberrant prothrombin precursor generated during hepatocyte malignant transformation, showed superior performance for HCC detection as compared to AFP [12,13]. Furthermore, the assessment of PIVKA-II showed promising results for the identification of HCC occurrence in patients with hepatitis C virus (HCV)/hepatitis B virus (HBV)-related cirrhosis [14,15,16,17]. One important limitation to the implementation of PIVKA-II in the surveillance of HCC is the uncertainty on which should be the cut-off value to consider. To date, several cut-off values have been proposed, 40 mAU/mL being the most widely adopted [18]. However, cut-off values should be selected based on the intended purpose (i.e., HCC surveillance, response to treatment, recurrence), taking into account local epidemiology and the various methods adopted for PIVKA-II determination.

The aim of the present study was to identify the best cut-off value of PIVKA-II for the surveillance of patients with liver cirrhosis at risk of developing HCC.

## 2. Materials and Methods

### 2.1. Patients

This retrospective, multicenter study included patients with liver cirrhosis recruited at the liver clinics of the Città della Salute e della Scienza di Torino—Molinette Hospital and the AOU Maggiore della Carità” of Novara from November 2012 to January 2022.

Inclusion criteria were age ≥18 years, diagnosis of liver cirrhosis, serum sample availability for PIVKA-II measurement, and signed written informed consent. Patients administered with oral anticoagulants were excluded from the study.

For all patients included in the present study, we collected baseline demographic (age and gender), clinical (liver disease etiology and severity of liver cirrhosis), and biochemical features, including alanine aminotransferase (ALT), aspartate aminotransferase (AST), platelet count, albumin, and total bilirubin values. For patients with a diagnosis of HCC, we collected data regarding nodules’ size and number and performance status. Baseline variables correspond to the time of serum sample collection. For patients with cirrhosis but without HCC, we collected clinical data since the last follow-up (FU) or HCC development.

The diagnosis of liver cirrhosis was achieved by histologic examination, or by liver elastrography (FibroScan^®^, Echosens™, Paris, France) and clinical and/or US features of portal hypertension [19,20]. HCC was diagnosed by histology or by imaging methods (multiphasic computed tomography or dynamic contrast-enhanced magnetic resonance), showing hypervascularity in the late arterial phase and washout on the portal venous and/or delayed phases [21]. HCC was classified according to the Barcelona Clinic Liver Cancer (BCLC) staging system [21].

### 2.2. Measurement or Serum PIVKA-II

Serum samples were collected from all patients included in the study and stored at −80 °C until analysis. PIVKA-II was measured by chemiluminescence enzyme immunoassay (CLEIA) (Lumipulse^®^ G PIVKA-II, Fujirebio Inc., Tokyo, Japan) on the fully automated Lumipulse^®^ G600 II analyzer (Fujirebio Inc., Tokyo, Japan) following the manufacturer’s recommendations. Lumipulse^®^ G PIVKA-II assay is characterized by precision <4.4%, and limit of detection = 0.075 mUA/mL.

### 2.3. Statistical Analysis

Categorical variables were reported as number (*n*) and frequencies (%), while quantitative continuous variables were reported as median and interquartile range (IQR). Data distribution was analyzed by D’Agostino-Pearson test. The Chi-squared test (χ^2^) test was used to analyze the distribution of categorical variables among the different groups. The Mann-Whitney and Kruskal-Wallis tests were used to compare continuous variables between two or more independent groups, respectively. Spearman correlation analysis was used to assess the correlation between continuous data; the strength of correlation was reported as *r_s_* and 95% confidence interval (CI).

The diagnostic accuracy of PIVKA-II was evaluated by receiver operating characteristic (ROC) curve analysis. PIVKA-II performance was reported as area under the curve (AUC); the corresponding sensitivity (Se), specificity (Sp), positive (+LR), and negative likelihood ratios (–LR) were calculated at different PIVKA-II cut-offs.

Cox regression analysis was used to evaluate the association between baseline PIVKA-II serum values and the risk of HCC occurrence; the strength of the association was reported as hazard ratio (HR) and 95% CI. Survival curves were analyzed according to the Kaplan-Meier method.

We considered statistically significant a two tailed *p* value < 0.05. All the analyses were performed using MedCalc software version 20.1 (MedCalc bvba, Ostend, Belgium).

## 3. Results

### 3.1. Comparison of Clinical Features between Patients with and without HCC

Overall, 1187 patients with liver cirrhosis were enrolled in the study. Their principal characteristics are reported in Table 1. The median age was 64, 57–74 years; most patients were males (*n* = 718; 60.5%). The main underlying liver disease etiology was chronic HCV infection (*n* = 965; 81.3%).

At baseline, 205 out of 1187 (17.3%) patients had a diagnosis of HCC. Among patients with HCC, we observed a higher proportion of males (*n* = 166; 81.0%) as compared to those without tumor (*n* = 552; 56.2%) (*p* < 0.001). Furthermore, HCC patients displayed a more compromised liver function in comparison to those without tumor, showing low representation of Child-Turcotte-Pugh class A, lower platelet count, lower albumin values, and higher total bilirubin (all *p* < 0.001). Most of HCC patients had a diagnosis of early tumor (BCLC 0/A = 149; 72.7%); overall, 110 out of 205 (53.7%) had a monofocal HCC.

### 3.2. Diagnostic Accuracy of PIVKA-II for HCC Detection

**Figure 1:**
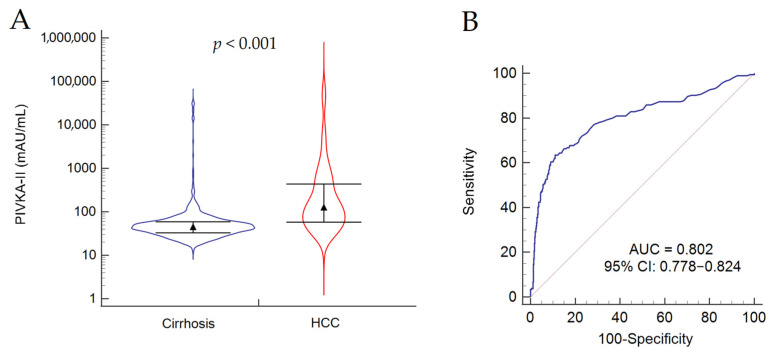
PIVKA-II serum values in patients with cirrhosis and in those with HCC (**A**) and corresponding ROC curve for the discrimination between patient with and without HCC at baseline (**B**). PIVKA-II values in patients with cirrhosis without HCC: median 45 mAU/mL (IQR 33–59). PIVKA-II values in patients with HCC: median 128 mAU/mL (IQR 58–435). Abbreviations: AUC, area under the curve; CI, confidence interval; FU, follow-up; HCC, hepatocellular carcinoma; PIVKA-II, protein induced by vitamin K absence or antagonist II.

The serum values of PIVKA-II were significantly higher (*p* < 0.001) in patients with HCC in comparison to those without tumor (*p* < 0.001) (Figure 1A). Serum PIVKA-II values showed no correlation with age (*r_s_* = 0.016, 95% CI −0.041–0.073, *p* = 0.590), while they were poorly correlated with biochemical features, such as ALT (*r_s_* = 0.163, 95% CI 0.106–0.220, *p* < 0.001), AST (*r_s_* = 0.163, 95% CI 0.105–0.220, *p* < 0.001), platelet count (*r_s_* = −0.176, 95% CI −0.233–−0.119, *p* < 0.001), albumin (*r_s_* = −0.089, 95% CI −0.150–−0.026, *p* < 0.001), and total bilirubin (*r_s_* = 0.100, 95% CI 0.041–0.160, *p* < 0.001) (Figure S1). Overall, serum PIVKA-II values were significantly higher in males in comparison to females (53, 39–83 mAU/mL vs. 40, 31–58 mAU/mL, respectively; *p* < 0.001); the same held true when we analyzed only patients without HCC at baseline (40, 30–55 mAU/mL in females vs. 48, 38–64 mAU/mL in males; *p* < 0.001). Conversely, when we analyzed only patients with HCC at baseline, no differences were observed according to sex (110, 50–268 mAU/mL in females vs. 129, 58–460 mAU/mL in males; *p* = 0.436) (Figure S2).

By ROC curve analysis, we observed a good diagnostic accuracy for the discrimination between patients with cirrhosis (*n* = 982) and those with HCC (*n* = 205) (AUC = 0.802, *p* < 0.001) (Figure 1B).

The cut-off maximizing both Se and Sp was 83 mAU/mL (Youden index; Se = 63%; Sp = 89%). However, for the purpose of surveillance, the cut-off that maximized Se (80%) without reasonable reducing Sp (64%) was 50 mAU/mL (Table 2); the percentage of cases correctly classified was 83%.

**Figure 2:**
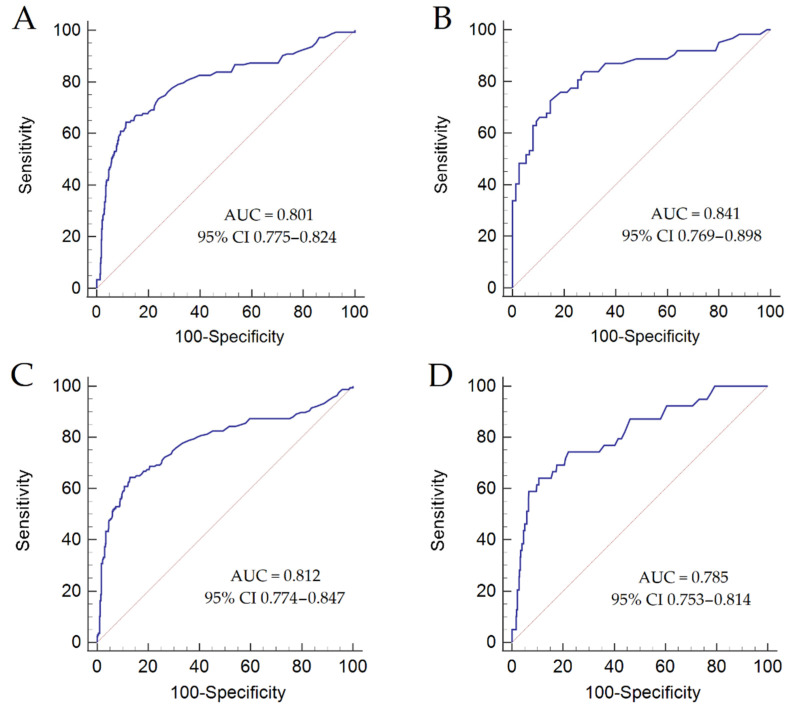
PIVKA-II ROC curves for the detection of HCC in patients with viral (**A**) and non-viral etiology (**B**), and according to sex ((**C**) = males; (**D**) = females). Abbreviations: AUC, area under the curve; CI, confidence interval.

Then, we performed a subgroup analysis according to the etiology of liver disease and sex. The diagnostic accuracy of PIVKA-II for HCC detection in patients with viral etiology was AUC = 0.801 and in those with non-viral etiology was AUC = 0.841 (DeLong test, *p* = 0.350) (Figure 2A,B). The cut-off of 50 mAU/mL showed Se = 81%, Sp = 60%, +LR = 2.08, and –LR = 0.29 in patients with viral etiology, and Se = 77%, Sp = 77%, +LR = 3.42, and −LR = 0.29 in patients with non-viral etiology.

The diagnostic accuracy of PIVKA-II for HCC detection in females was AUC = 0.812, while in males it was AUC = 0.785 (DeLong test, *p* = 0.556) (Figure 2C,D). The cut-off of 50 mAU/mL showed Se = 74%, Sp = 70%, +LR = 2.52, and −LR = 0.36 in females, and Se = 83%, Sp = 55%, +LR = 1.82, and −LR = 0.32 in males.

### 3.3. Stratification of the Risk of HCC Development According to PIVKA-II

During a median follow-up (FU) of 34.6 (11.4–43.7) months, 118 out of 982 (12.0%) patients with cirrhosis developed HCC. Remarkably, the median PIVKA-II serum values resulted significantly higher in patients that developed HCC (*n* = 118) in comparison to patients HCC-free at last FU (*n* = 864) (55, 36–78 mAU/mL vs. 44, 33–57 mAU/mL, respectively; *p* < 0.001).

**Figure 3:**
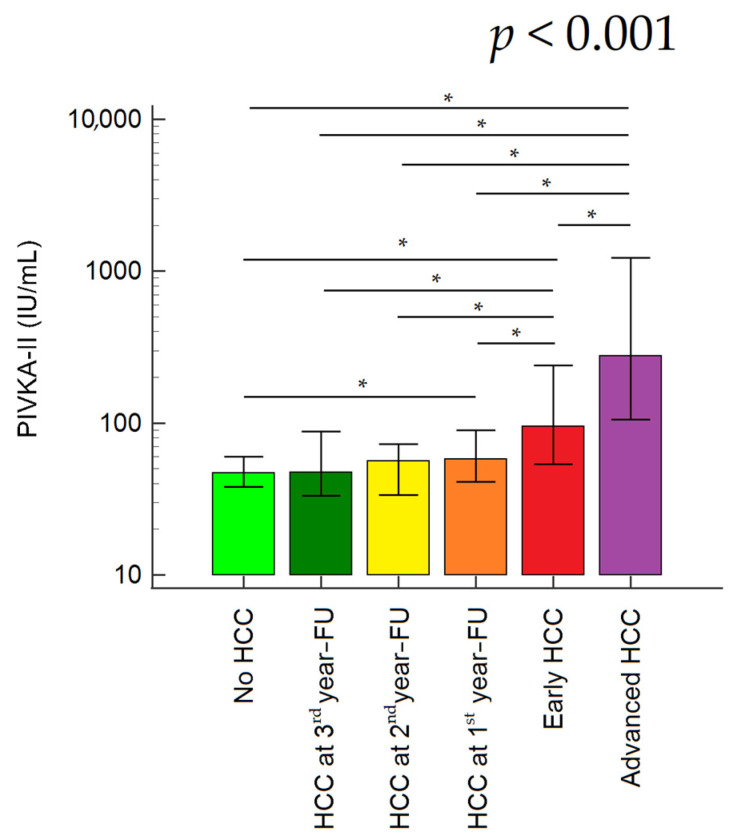
Serum PIVKA-II values in different subgroups of patients. The lowest PIVKA-II values were observed in patients with cirrhosis without HCC occurrence during the FU (47, 38–60 mAU/mL), followed by patients with HCC occurrence during the third (48, 34–89 mAU/mL) and second year of FU (56, 34–73 mAU/mL). PIVKA-II values significantly increased in patients with HCC occurrence during the first year of FU (58, 41–90 mAU/mL), followed by patients with early HCC at baseline (95, 54–240 mAU/mL), and further increased in patients with advanced HCC at baseline (277, 106–1221 mAU/mL). * *p* value < 0.05 (Mann-Whitney test). Abbreviations: FU, follow-up; HCC, hepatocellular carcinoma; AUC, area under the curve; CI, confidence interval.

Furthermore, serum PIVKA-II values distinctly increased according to the time of HCC occurrence and tumor stage; serum PIVKA-II increased stepwise from patients with cirrhosis who did not develop HCC during the FU, to those with early HCC occurrence, and among patients with HCC at baseline further increased from those with early HCC (BCLC = 0/A) to patients with advanced tumor (BCLC = B/C/D) (Kruskal-Wallis test; *p* < 0.001) (Figure 3).

**Figure 4:**
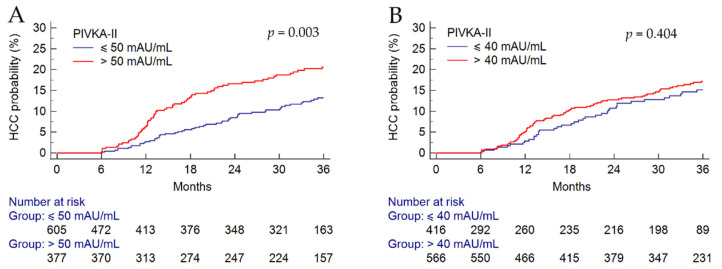
HCC incidence in patients with cirrhosis according to baseline serum PIVKA-II > 50 mAU/mL (**A**) and >40 mAU/mL (**B**). Abbreviations: HCC, hepatocellular carcinoma; PIVKA-II, protein induced by vitamin K absence or antagonist II.

Finally, in patients with cirrhosis but without HCC at baseline (*n* = 982), PIVKA-II > 50 mAU/mL was significantly associated with an increased risk of HCC development during the FU (HR = 1.74, 95% CI 1.21–2.51; *p* = 0.003). Consistently, the cumulative incidence of HCC in the 605 patients with baseline PIVKA-II ≤ 50 mAU/mL was significantly higher as compared to HCC occurremce in the 377 patients with baseline PIVKA-II > 50 mAU/mL (8.6% vs. 17.5%, Log-rank test, *p* = 0.003) (Figure 4A). Conversely, we did not observe an association between HCC occurrence and a PIVKA-II > 40 mAU/mL (HR = 1.17, 95% CI 0.80–1.73; *p* = 0.404); indeed, 38 out of 416 (9.1%) patients with baseline PIVKA-II ≤ 40 mAU/mL developed HCC as compared to 80 out of 566 (14.1%) patients with baseline PIVKA-II > 40 mAU/mL (Log-rank test, *p* = 0.404) (Figure 4B).

## 4. Discussion

In the present study, we confirmed the good diagnostic accuracy of PIVKA-II for HCC detection among patients with liver cirrhosis, and we identified the value of 50 mAU/mL as appropriate PIVKA-II cut-off for the surveillance of patients with cirrhosis at risk of HCC development.

To date, the use of serum biomarkers for the surveillance of patients at risk of HCC development is still a matter of debate within the scientific and medical community. Even international guidelines are not uniformly aligned in their recommendations. The European Association for the Study of the Liver recommends US only [7] due to the suboptimal cost-effectiveness of circulating biomarkers. The American Association for the Study of Liver Diseases recommends biannual surveillance using US, with or without AFP [21]. Conversely, Eastern guidelines endorse the use of serum biomarkers [22,23]; the recommended cut-off value of PIVKA-II is 40 mAU/mL. In our study, we observed that a PIVKA-II cut-off value of 50 mAU/mL yielded good Se (80%) and acceptable Sp (64%); in our population, lowering the cut-off value to 40 mAU/mL drastically reduced the Sp (42%) with only marginal improvement of Se (87%). From a practical perspective, the implementation of a lower cut-off value would result in an increase in false positive cases decreasing the efficiency of the surveillance program.

Interestingly, in patients with cirrhosis without HCC at baseline, we observed slightly higher serum PIVKA-II values in males as compared to females, while no difference was observed among patients with HCC. Consistent with our data, a previous multicenter study, including 892 healthy subjects from seven regional centers in China, pointed out different median PIVKA-II values according to sex, with higher values in males compared to females [24]. Despite the role of sex on vitamin K, homeostasis has not been fully characterized in humans, pre-clinical studies pointed out a specific effect of sex hormones on vitamin K concentration through the regulation of the expression and function of key metabolic enzymes involved in vitamin K metabolism [25]. Taken together, these results warrant further studies to specifically investigate the impact of sex on PIVKA-II serum levels and thus the potential need for sex-specific PIVKA-II cut-offs.

A considerable number of case-control studies investigating the diagnostic accuracy of PIVKA-II assessed by CLEIA methods for the discrimination between patients with and without HCC indicated a wide spectrum of cut-off values ranging from 32 mAU/mL to 200 mAU/mL, with the corresponding Se = 52–91% and Sp = 66–93% [26,27,28,29]. Conversely, only few studies investigated the value of PIVKA-II measurement in patients at risk of HCC development. In Caucasian patients with HBV-related cirrhosis under long-term treatment with nucleos(t)ide analogues, a PIVKA-II > 48 mAU/mL resulted in Se = 64% and Sp = 91% for the HCC detection; remarkably, 50% of the patients already showed PIVKA-II > 48 mAU/mL 12 months before HCC detection by imaging [14]. Similarly, in Asian patients with HBV-related cirrhosis, PIVKA-II > 50 mAU/mL at the time of antiviral therapy-induced virologic remission was associated with a greater risk of HCC (HR = 2.46, 95%CI 1.35–4.49) [30]. In patients with HCV-related cirrhosis treated with direct acting antivirals, an end-of-treatment PIVKA-II value > 41 mAU/mL resulted significantly associated with the risk of HCC occurrence during the FU; the estimated four-year HCC incidence was 24% in patients with PIVKA-II > 41 mAU/mL, as compared to the 2% of those with PIVKA-II ≤ 41 mAU/mL (*p* < 0.001). At HCC detection, the cut-off granting the best PIVKA-II performance was 47 mAU/mL (AUC = 0.780; Se = 76% and Sp = 79%) [16]. Finally, we previously observed on a cohort of 200 patients with viral-related cirrhosis that PIVKA-II > 55 mAU/mL was significantly and independently associated with HCC development (HR = 1.99, 95% CI 1.25–3.19, *p* = 0.004) [17]. Taken together, these results are in agreement with the findings of the present study. Possibly, differences in study design (prospective vs. retrospective), patients’ enrollment (consecutive vs. non-consecutive patients), and underlying liver disease etiology [12], may have accounted for the minimal discrepancies among the different cut-offs proposed.

Some limitations need to be acknowledged. Firstly, the retrospective nature of the study hindered a comprehensive characterization of the patients enrolled and the complete assessment for potential confounding factors. Secondly, we did not perform a cost-effectiveness analysis. Lastly, since the patients included in our study were not consecutively enrolled, data on the incidence of HCC during the FU and the prevalence of chronic liver diseases etiology cannot be fully contextualized into real-life clinical practice.

## 5. Conclusions

The assessment of serum PIVKA-II showed a good performance for HCC detection; a cut-off value > 50 mAU/mL provided a reasonable sensitivity and specificity for the discrimination of patients with HCC and those without tumor.

Finally, PIVKA-II appeared useful to stratify the risk of HCC development in patients with cirrhosis under surveillance; future prospective studies are needed to define personalized surveillance strategies according to patients’ individual risk.

## Figures and Tables

**Table 1:** Demographic, clinical, and biochemical characteristics of the patients enrolled in the study.

Variables	Overall	Cirrhosis	HCC	*p* Value
Number of patients	1187	982	205	
Age (years)	65 (57–76)	64 (57–76)	67 (61–77)	0.142
Sex (M/F)	718/469	552/430	166/39	<0.001
Liver disease etiology HCV HBV ^1^ Dysmetabolic/other	965 (81.3%) 85 (7.2%) 137 (11.5%)	859 (87.5%) 48 (4.9%) 75 (7.6%)	106 (51.7%) 37 (18.0%) 62 (30.3%)	<0.001
Child-Turcotte-Pugh score A	1088 (91.7%)	922 (93.9%)	171 (83.4%)	<0.001
ALT (U/L)	23 (17–41)	22 (16–35)	46 (28–81)	<0.001
AST (U/L)	28 (21–42)	26 (21–37)	58 (35–94)	<0.001
Platelets (×10^9^/L)	130 (86–180)	139 (92–185)	102 (68–133)	<0.001
Albumin (g/dL)	4.2 (3.9–4.5)	4.3 (4.0–4.6)	3.8 (3.2–4.2)	<0.001
Total bilirubin (mg/dL)	0.8 (0.6–1.1)	0.8 (0.6–1.1)	1.2 (1.1–1.3)	<0.001
HCC nodules (1/2/3/>3)			110/40/36/19	
Size of major HCC nodule (mm)			23 (17–36)	
BCLC (0/A/B/C/D)			38/111/43/10/3	

^1^ Nine patients had HDV/HBV co-infection. Continuous variables were reported as median and IQR, while categorical variables as number and percentage. Abbreviations: ALT, alanine aminotransferase; AST, aspartate aminotransferase; F, female; HCC, hepatocellular carcinoma; HBV, hepatitis B virus; HCV, hepatitis C virus; IQR, interquartile range; M, male.

**Table 2:** Sensitivity, specificity, positive, and negative likelihood ratio of PIVKA-II values according to different cut-offs.

PIVKA-II Cut-Offs	Se	Sp	+LR	−LR
40 mAU/mL (widely used cut-off)	87%	42%	1.51	0.30
50 mAU/mL (maximizing Se)	80%	64%	2.18	0.32
65 mAU/mL (maximizing Sp)	68%	80%	3.46	0.40
83 mAU/mL (Yuden index)	63%	89%	5.71	0.41

Abbreviations: PIVKA-II, protein induced by vitamin K absence or antagonist II; Se, sensitivity; Sp, specificity; +LR, positive likelihood ratio; −LR, negative likelihood ratio.

## Data Availability

The data presented in this study are available upon request from the corresponding author.

## Supplementary Materials

The following supporting information can be downloaded at: https://www.mdpi.com/article/10.3390/biology12010094/s1, Figure S1: Correlation between serum PIVKA-II values and age (A), ALT (B), AST (C), platelet count (D), albumin €, and total bilirubin (F); Figure S2: Serum PIVKA-II values in males and females according to the diagnosis of HCC.
