# Supplementary material for: Identification of the Best Cut-Off Value of PIVKA-II for the Surveillance of Patients at Risk of Hepatocellular Carcinoma Development
Source: Biology (Basel). 2023 Jan 7;12(1):94. doi: 10.3390/biology12010094 (PMC9855902; doi:10.3390/biology12010094)
Supplement: Supplementary file 1 [file biology-12-00094-s001.zip › biology-2089715-supplementary.pdf]

biology

MDPI

Supplementary Material

Identification of the best cut-off value of protein induced by vitamin K absence or antagonist-II for surveillance of patients with cirrhosis at risk of hepatocellular carcinoma development

Gian Paolo Caviglia, Maria Lorena Abate, Giulia Troshina, Patrizia Carucci, Emanuela Rolle, Alessandra Risso, Michela Emma Burlone, Alice Albè, Martina Crevola, Emma Clara Musso, Chiara Rosso, Angelo Armandi, Antonella Olivero, Rosalba Minisini, Giorgio Maria Saracco, Elisabetta Bugianesi, Mario Pirisi, Alessia Ciancio, Silvia Gaia

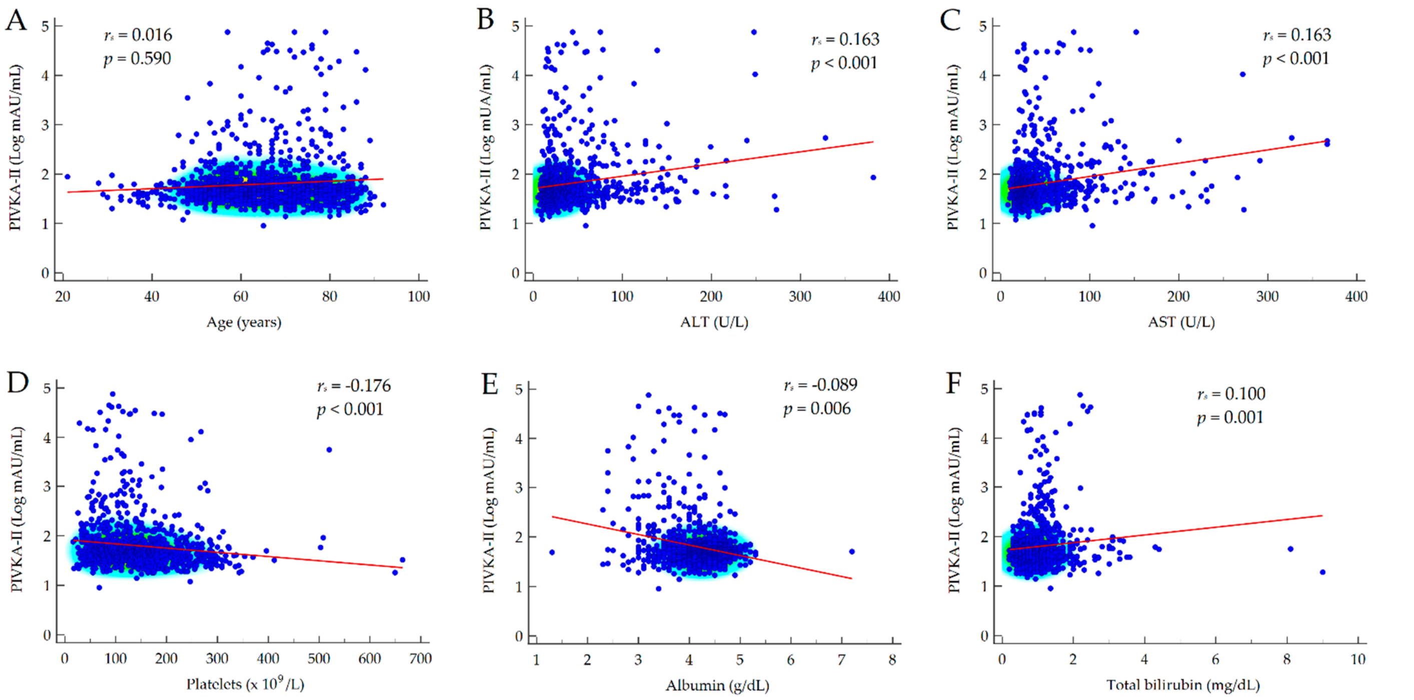

Figure S1. Correlation between serum PIVKA-II values and age (A), ALT (B), AST (C), platelet count (D), albumin (E), and total bilirubin (F). Correlation coefficients were calculated by Spearman rank correlation test. Abbreviations: alanine aminotransferase (ALT), aspartate aminotransferase (AST), protein induced by vitamin K absence or antagonist II (PIVKA-II).

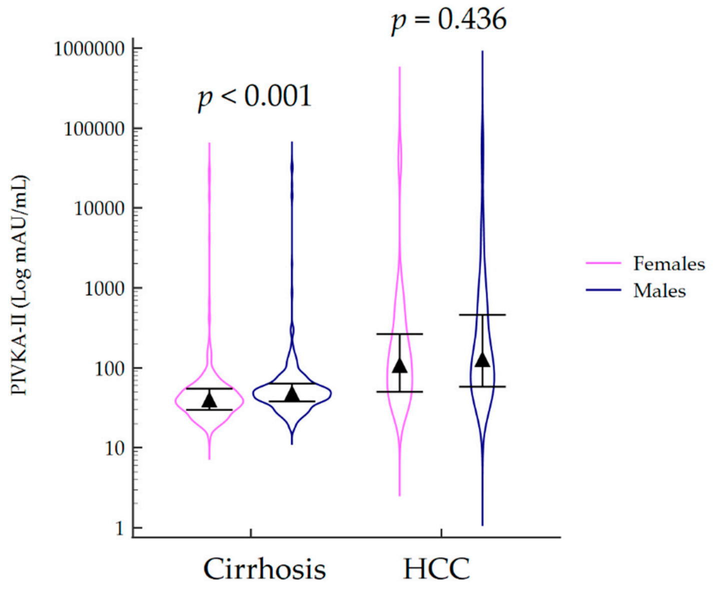

Figure S2. Serum PIVKA-II values in males and females according to the diagnosis of HCC. In patients with no HCC at baseline, median PIVKA-II serum values were 40 (30–55) mAU/mL in females and 48 (38–64) mAU/mL in males ($p < 0.001$), while in patients with a diagnosis of HCC, median serum PIVKA-II values were 110 (50–268) mAU/mL in females and 129 (58–460) mAU/mL in males ($p = 0.436$). Abbreviations: hepatocellular carcinoma (HCC), protein induced by vitamin K absence or antagonist II (PIVKA-II).
